# Reversible Aggregation of Molecular-Like Fluorophores Driven by Extreme pH in Carbon Dots

**DOI:** 10.3390/ma13163654

**Published:** 2020-08-18

**Authors:** Stefania Mura, Luigi Stagi, Robert Ludmerczki, Luca Malfatti, Plinio Innocenzi

**Affiliations:** Laboratorio di Scienza dei Materiali e Nanotecnologie, CR-INSTM, Dipartimento di Chimica e Farmacia, Università di Sassari, Via Vienna 2, 07100 Sassari, Italy; stmura@uniss.it (S.M.); lstagi@uniss.it (L.S.); ludmerczki@gmail.com (R.L.); luca.malfatti@uniss.it (L.M.)

**Keywords:** nanomaterials, carbon-dots, photoluminescence, spectroscopy

## Abstract

The origin of carbon-dots (C-dots) fluorescence and its correlation with the dots structure still lack a comprehensive model. In particular, the core-shell model does not always fit with the experimental results, which, in some cases, suggest a molecular origin of the fluorescence. To gain a better insight, we have studied the response of molecular-like fluorophores contained in the C-dots at extreme pH conditions. Citric acid and urea have been employed to synthesize blue and green-emitting C-dots. They show a different emission as a function of the pH of the dispersing media. The photoluminescence has been attributed to molecular-like fluorophores: citrazinic acid and 4-hydroxy-1H-pyrrolo[3,4-c]-pyridine-1,3,6-(2H,5H)-trione. 3D and time-resolved photoluminescence, ultraviolet–visible (UV–vis) spectroscopy, and dynamic light scattering have been used to determine the aggregation state, quantum yield and emission properties of the C-dots. The dependence of the C-dots blue and green components on the chemical environment indicates that the origin of fluorescence is due to molecular-like fluorophores.

## 1. Introduction

Carbon dots (C-dots) are luminescent materials whose potential applications span different fields: lasing, photocatalysis, chemical and biological sensing and bioimaging [1,2]. Many synthesis methods have been developed so far, in particular bottom-up routes based on the polymerization and carbonization of the molecular precursors [3]. Several syntheses employ citric acid as a precursor [4,5], which has the advantage of being highly biocompatible [6]. The quantum yield (QY) of the product is in general in the range of 5–15%, which is quite low in comparison to C-dots obtained with other precursors [7]. A better QY is achieved by adding a nitrogen-source as a precursor, such as urea [8,9]. In a recent work [10], we have reported the high fluorescence tunability C-dots synthesized by carbonization of citric acid and urea. A marked blue to green shift has been obtained as a function of the precursors molar ratio.

Although a substantial number of works have been already published on the topic, a controversial debate about the origin of C-dots fluorescence is still ongoing. Some authors attribute the C-dots emission properties to the functional groups on the dot surface, others to molecular-like fluorophores, which form during the thermal degradation process [11,12,13]. Conventionally, carbon dots can be divided into four classes [14]. Graphene quantum dots (GQDs) are small fragments of one or a few layers of graphene displaying a size-dependent fluorescence originating from quantum confinement effects. Carbon quantum dots (CQDs) present quantum confinement effects as well. CQDs have a graphitic core with several functional groups at the surfaces. Carbon nanodots (CNDs) typically do not show a well-defined crystal lattice and tend to display a molecular fluorescence, although many papers report discordant results [15,16,17]. Polymeric carbon dots (PCDs) derive from the temperature-induced aggregation and carbonization of polymers. This results in a carbon core functionalized by long polymeric chains [14].

A general concept of the core-shell model is frequently applied to describe the optical properties of C-dots. In this model, the overall optical emission of C-dots depends on two main contributions: the inner carbon core part in the form of sp^2^ conjugated frameworks, which emits at higher energies (blue region) [14,18] and the surface states. Identifying the origin of surface states has represented one of the main challenges for C-dots engineering so far. The surface states can be attributed to dangling bonds, nitrogen- and oxygen-containing functional groups, and sp^2^-sp^3^ carbons. On those sites rely the fluorescent emissions in the green and red regions [19]. After absorption of high energy photons (ultraviolet (UV)-blue) by the inner part of the C-dots, the excitons can be captured by the edge states and can contribute to the excitation of the surface [19,20]. According to this model, the emission related to the edge states is strongly affected by the chemical surroundings.

An alternative explanation of the C-dot emission is based on the formation of molecular-like fluorophores. Fluorescent derivatives of the initial C-dots precursors form during the thermal degradation process. More specifically, the carbonization of citric acid combined with N-sources (such as urea or ammines) leads to the formation of pyridone derivatives, which are typically fluorescent [21,22]. The presence of molecular-like fluorophores gives different photophysical properties depending on the accessibility of these molecules to the surroundings. A well-isolated molecule, embedded in a dense matrix, should not be affected by changes in the polarity or pH of the solvents. On the other hand, weakly-entrapped fluorophores should deeply change their emission depending on the interaction with the solvent and the aggregation state.

In citric and urea derived C-dots, different studies have identified the origin of the blue and green luminescence with the formation of citrazinic acid and its derivatives [23] or 4-hydroxy-1H-pyrrolo[3,4-c]-pyridine-1,3,6-(2H,5H)-trione [24] (HPPT), respectively. How these fluorophores are sensitive to the external environment is, however, not clear. It has been reported that the variation of pH may induce fluorescence tuning and quenching [12,25].

Although this effect could provide new insights into the interaction of C-dots with the chemical environment and, in a second place, information about the structure, the mechanism of pH-induced modulation of the luminescence is still unknown. Recently, a paper from Tan and co-authors has reported that C-dots prepared by citric and folic acid show an emission that is pH dependent [26]. Those C-dots, however, show a crystalline form with a graphitic core. To the best of our knowledge, there are no papers focused on the study of amorphous C-dots, whose fluorescence is mainly due to molecular fluorophores.

In this work, we have investigated the optical properties of blue- and green-emitting C-dots under extreme pH conditions. In more detail, we have analyzed the effect of sulfuric acid and sodium hydroxide on C-dots synthesized at high (1:2) and low (1:25) citric acid/urea molar ratios, whose optical properties in water and ethanol were already known and assigned to specific fluorophore molecules. The response at extreme pH of citric acid-urea C-dots has allowed us to study in depth the molecular origin of the C-dots fluorescence, which is of paramount importance for the synthesis and engineering of carbon-based nanomaterials with high efficiency and controlled fluorescent emission.

## 2. Materials and Methods

### 2.1. Chemicals and Reagents

Citric acid monohydrate (purity 99.9%, Honeywell Fluka^TM^, Monza, Italy), urea for electrophoresis (purity 98%, Merck Sigma-Aldrich, Milan, Italy), sodium hydroxide anhydrous (pellets, Carlo Erba, Milan, Italy), sulfuric acid (96% w/w, Carlo Erba, Milan, Italy) and water (milli-Q) were used as received without further purification. Whatman GD/X syringe filters (Fisher Scientific, Rodano, Italy) made of a porous polyvinylidene difluoride (PVDF) membrane having a pore size of 0.20 μm were used for filtration.

### 2.2. Synthesis of Citric Acid-Urea C-Dots

C-dots were prepared by following an already published synthesis [10]. Briefly, blue- and green-emitting C-dots were prepared by using two precursors: citric acid (CA) and urea, with molar ratios 1:2 (sample CU2) and 1:25 (sample CU25), respectively. CA and urea were put in an open round bottomed flask, dissolved in 10 mL of mQ water, and heated in an oil bath at 190 °C for 2 h. To remove large aggregates from the C-dots, the black powders obtained from the process were at first dissolved in water and then centrifuged at 12,000 rpm for 20 min. After this, the supernatants were filtered with a syringe filter. Finally, the water solutions containing the CU2 and CU25 samples were evaporated to obtain a solid product. The optical properties of CU2 and CU25 were investigated in water as a reference at the concentration of 1, 10, and 100 mg·L^–1^. The corresponding pH values varied from 6.3 (1,10 mg L^−1^) to 6.0 (100 mg L^−1^) for CU2 and 6.3 (1,10 mg L^−1^) to 6.2 (100 mg L^−1^) for CU25, respectively.

### 2.3. Study of the Fluorescence of C-Dots Dissolved in Strong Acid and Base

CU2 and CU25 in the form of powders were dissolved in solutions of sulfuric acid at different dilutions (40, 20, and 10% (*v/v)* aqueous solutions). In particular, the CU2 C-dots dissolved in sulfuric acid at pH 1 (10% dilution) have been compared with the C-dots dissolved in aqueous sodium hydroxide solutions at pH 14, with a final concentration of 10 mg L^−1^. C-dots in water were used as a reference and were analyzed at a concentration of 1 mg L^−1^ for ultraviolet–visible (UV–Vis) and for photoluminescence (PL) analysis. A neutralization reaction was carried out adding NaOH pellets until reaching pH 7 to a solution of CU2 C-dots in H_2_SO_4_ 10% with a concentration of 10 mg L^−1^. 0.1 and 1 mg L^−1^ was considered for neutralization experiments, as well.

### 2.4. Characterization Methods

UV–Vis measurements were performed in absorbance, using a Nicolet Evolution 300 spectrophotometer (ThermoFisher Scientific, Waltham, Massachusetts, USA) from 200 to 600 nm.

Fluorescence spectroscopic measurements were taken using a Horiba Jobin Yvon FluoroMax-3 spectrofluorometer (Rome, Italy). For three-dimensional mapping (excitation-intensity-emission), a 450 W Xenon lamp was used as the excitation source. The maps were collected with an excitation range of 200–700 nm and an emission range of 200–700 nm with a 2 nm slit for excitation and emission in water and 5 nm slit for acidic and basic solutions. Solutions of C-dots at 100 mg L^−1^ were analyzed with the particle size analyzer DLS (dynamic light scattering) Horiba LB-550. Time-resolved fluorescence measurements (TR) in the nanosecond time domain were performed using the TBX picosecond detection module connected to a Nanolog spectrometer (Horiba Jobin Yvon, Rome, Italy). The samples were excited with a 340 nm light-emitting diode (LED) source (1 MHz repetition rate and pulse width <1.2 ns) and with a 405 nm laser diode (1 MHz repetition rate and pulse width <0.2 ns). Data were collected immediately after laser decay. The acquisition time resolution is 0.11 ns.

Absolute photoluminescence quantum yield (AQY) measurements were performed using the quanta-ϕ (Horiba) integrating sphere accessory, attached to the “NanoLog” spectrofluorometer (Rome, Italy). Water was used as a blank reference exciting at 350 nm for blue-emitting dots and 420 nm for green-emitting C-dots.

Transmission electron microscopy (TEM) images were acquired by using a FEI TECNAI 200 microscope (ThermoFisher Scientific, Waltham, MA, USA) working with a field emission gun operating at 200 kV. The samples were dropped onto a carbon-coated grid and dried for observations.

## 3. Results and Discussion

The optical response of C-dots prepared from citric acid and urea in acid and alkaline solutions have been systematically studied to reveal the dependence of fluorescence properties on the variations of the fluorophore protonation and deprotonation. Water-dissolved C-dots were used as a reference.

### 3.1. C-Dots Reference Samples

Transmission electron microscopy (TEM), UV–Vis absorption and photoluminescence spectra were used to characterize the CU2 and CU25 samples and confirm their respective morphological and spectral features. Transmission electron microscopy images of as-prepared C-dots (Figure S1**)** confirm that both samples do not show a crystalline structure in accordance with our previous work [10].

**Figure 1:**
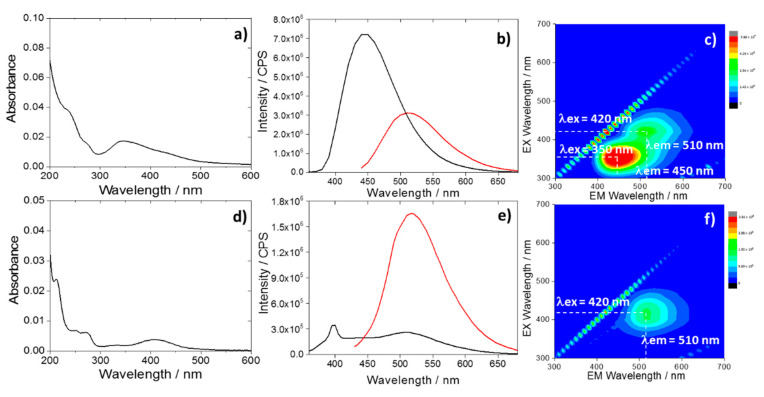
Ultraviolet–visible (UV–Vis) spectra of the CU2 (**a**) and CU25 (**d**) C-dots dispersed in water at a concentration 1 mg L^−1^; (**b**) and (**e**) photoluminescence (PL) emissions of CU2 and CU25 C-dots in water (1 mg L^−1^) with excitations at 350 nm (black) and 420 nm (red). The peak at 398 nm under excitation at 350 nm is due to the Raman signal of water. (**c**) and (**f**) 3D photoluminescence spectrum (excitation (y-axis), emission (x-axis), intensity (false colors scale)) of the CU2 and C25 C-dots in water (C-dots concentration = 1 mg L^−1^).

The UV–Vis absorption spectrum of the CU2 C-dots in water (Figure 1a) shows the characteristic UV absorption bands peaking at 234 nm and 345 nm. Citric acid and urea derived C-dots are known to exhibit these bands [12]. Nevertheless, they are also detected in the absorption spectrum of citrazinic acid dissolved in water [27]. Correspondingly, the CU2 C-dots absorption spectrum is mainly identified by molecular origin, as a result of the formation of citrazinic acid molecule by citric acid and urea reaction at high temperature (190 °C).

The CU2 C-dots are characterized by a blue fluorescence (Figure 1b,c) peaking at 450 nm, which corresponds to the characteristic citrazinic acid emission in water [27]. The C-dots show also a weaker green component assigned to the formation of a fluorophore identified with HPPT. The presence of this compound has been already observed in C-dots synthesized by citric acid and urea with an excess of urea and in anhydrous conditions [24].

Green emitting C-dots (CU25) were synthesized by increasing the urea content, according to a previous work [10]. The CU25 UV–Vis spectra (Figure 1d) display the UV absorption bands that are attributed to π-π* of HPPT [24]. The latter is already observed in CU2 dots. The UV–Vis absorption bands peaking at 425 nm (CU2) and 415 nm (CU25) can be attributed to the formation of aggregates of molecular-like fluorophores into the carbon dots. This peculiar optical response of citrazinic acid has also been observed in another experiment by Reckmeier et al. [2]. The origin of the green emission for CU25 C-dots was attributed to the formation of the HPPT molecular compounds. The 3D spectra of CU25 dots (Figure 1f) mainly show a single-emission pattern peaking at 510 nm (λ_ex_ = 420 nm) (Figure 1e).

Therefore, two main emissions, with maxima in the blue (λ_ex_ = 350 nm, λ_em_ = 450 nm) and green (λ_ex_ = 420 nm, λ_em_ = 510 nm) regions (Figure 1c), characterize the CU2 C-dots fluorescence spectra in water. The blue emission is the most intense (Figure 1b). By contrast, the green component results were enhanced in CU25 (Figure 1e), for the excess of urea.

### 3.2. Effect of Sulfuric Acid

The CU2 C-dots have been dispersed in aqueous solutions of sulfuric acid using different concentrations (40, 20, and 10% (*v/v)*).

**Figure 2:**
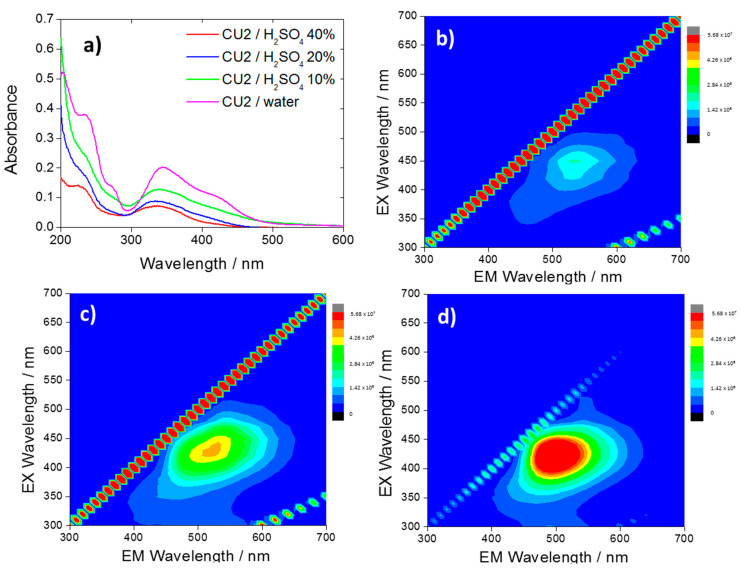
(**a**) UV–Vis spectra of CU2 C-dots in water (10 mg L^−1^, violet) and in sulfuric acid 10% (green), 20% (blue), 40% (red), and 3D photoluminescence spectra (excitation (y-axis), emission (x-axis), intensity (false colors scale)) of the CU2 C-dots in H_2_SO_4_, 40% (**b**); 20% (**c**) and 10% (**d**) C-dots concentration = 10 mg L^−1^.

By dissolving the CU2 C-dots in sulfuric acid 40% *(v/v)* (5.31 M) (Figure 2b), a quenching of the blue fluorescence was observed while the green one was still detected (intensity of 2 × 10^6^ CPS). The dissociation maximum of H_2_SO_4_ (*aq*) occurs between 5 M and 7 M where the highest concentration of protons and counter-ions in the solution can be observed [28]. This maximum acid concentration corresponds to a solution of sulfuric acid 40% *(v/v)*. It is worth noting that the citrazinic acid in analogous acidic conditions undergoes a luminescence quenching (Figure S2). This correspondence supports the hypothesis that the blue emission is originated by citrazinic acid and its derivatives.

After reaching the dissociation maximum (Figure 2b) (40%), a dilution of the acid to 20 and 10% (Figure 2c,d) causes an increase of the green fluorescence and a blue shift of the maximum from 530 to 500 nm. The UV–Vis spectra (Figure 2a) show that lowering the pH causes a decrease in the absorbance intensity.

The results suggest the presence of two fluorophores, which have specific optical responses to a change of the chemical environment. In a recent work [25] it has been shown that the citrazinic acid can form aggregates with a weak green emission, if dissolved in acidic solutions (Figure S2b). This phenomenon is explained with the protonation of the citrazinic acid molecules, associated with a blueshift and a weakening of the band at 275 nm. In accordance with the citrazinic acid response, CU2 C-dots exhibit the disappearance of the 275 nm band as an effect of the protonation. As illustrated in a recent article by Sakar et al. [29] the most stable specie of the citrazinic acid in strong acidic conditions is the imine form with the protonated carbonyl-, carboxyl- and nitrogen groups. In strong acidic conditions, the aggregation is favored due to the formation of intermolecular H-bonds and π-π stacking of the pyridone units [2].

**Figure 3:**
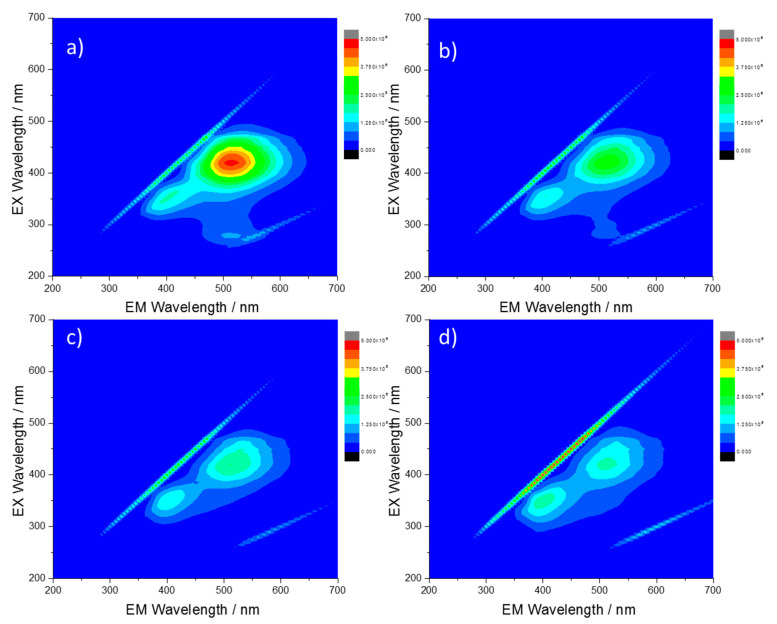
Three-dimensional photoluminescence spectra (excitation (y-axis), emission (x-axis), intensity (false colors scale)) of the CU25 C-dots in H_2_SO_4_, (**a**) 1%, (**b**) 10%, (**c**) 20% and (**d**) 40%. C-dots concentration = 10 mg L^−1^.

The emission of CU25 C-dots is differently affected by the solution acidity (Figure 3). At increasing acidity concentration, we observe both a decrease of the green contribution and the blue component (attributed to the residual amount of citrazinic acid). The PL response confirms the assumption of a different emission source for the CU25 sample, i.e., HPPT fluorophore.

### 3.3. Reversibility of the Quenching Effect

**Figure 4:**
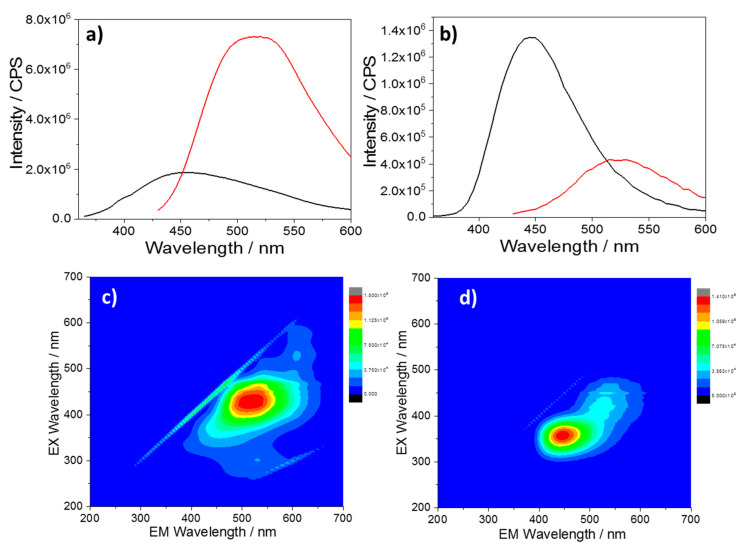
PL emissions of CU2 C-dots (**a**) in sulfuric acid (10%) with excitation at 350 nm (black) and 420 nm (red) and (**b**) after neutralization with NaOH pellets with excitation at 350 nm (black) and 420 nm (red). (**c**) and (**d**) PL maps of the same solutions in acid and after neutralization (C-dots concentration = 10 mg L^−1^).

To verify if the emission quenching is reversible, CU2 C-dots dissolved in sulfuric acid (10%) have been neutralized with NaOH and the 3D spectra have been recorded. Figure 4 shows the PL and the 3D fluorescence maps of C-dots upon excitation at 350 (black) and 420 nm (red) in acid and after neutralization. As a result of the neutralization, an intense blue emission appears, and, correspondingly, the green contribution decreases. A broadening of the green emission nm is also visible. This effect is attributed to the merging of the PL with the water Raman vibration, which is located at 491 nm under excitation at 420 nm. To confirm this attribution, we have measured the CU2 sample at lower concentrations (Figure S3). The PL spectra show a strong contribution of the Raman signal of water which becomes prominent as the CU2 concentration decreases. The reversibility of the quenching effect suggests that aggregation is a reversible process and the pristine emissions of the CU2 sample can be easily restored (see Figure 1b).

### 3.4. C-Dots in Highly Basic Solutions

**Figure 5:**
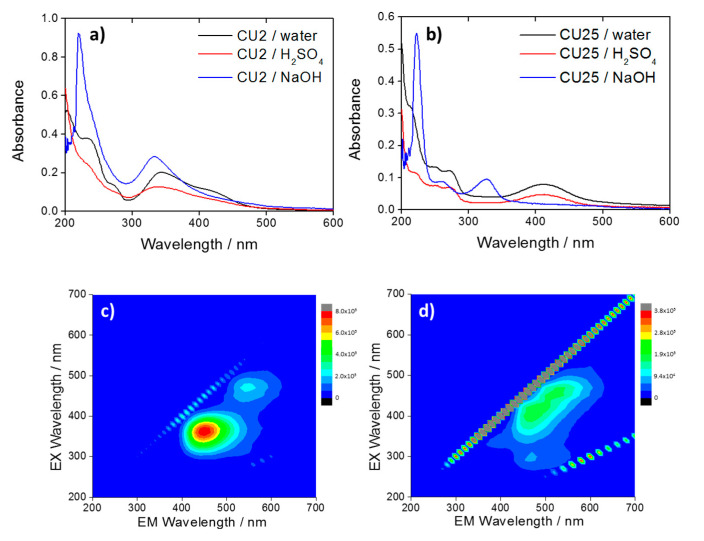
UV–Vis spectra of (**a**) CU2 and (**b**) CU25 in water (black) and in H_2_SO_4_ pH 1 (red) and NaOH pH 14 (blue). 3D photoluminescence spectra (excitation (y-axis), emission (x-axis), intensity (false colors scale) of **(c**) CU2 C-dots and (**d**) CU25 C-dots in NaOH. C-dots concentration = 10 mg L^−1^.

Figure 5 shows the absorption spectra of CU2 (Figure 5a) and CU25 (Figure 5b) in water (black), acid (red), and basic (blue) solutions. The overall spectra of both CU2 and CU25 in basic conditions appear very similar. The visible component at λ > 400 nm decreases for both samples at pH 14, up to a complete disappearance for CU25 dots. A blue shift of the 345 nm band in water is also observed for CU2 dots. Moreover, the rise of a new one at 328 nm is detected in the CU25 sample.

At pH 14, the molecular-like fluorophores are in a de-protonated form [25,28] which inhibits the formation of hydrogen bonds between fluorophores and avoids aggregation. This is in agreement with the disappearance of the absorption bands at 425 nm for CU2 (Figure 5a) and 410 nm for CU25 (Figure 5b) attributed to the aggregates [2].

The analysis of the CU2 3D PL map (Figure 5c) in NaOH (*aq*) shows two different residual bands, which can be attributed to citrazinic acid for the blue emission and, in a small amount, to HPPT for the green component. The CU25 3D PL map shows similar emissions in the blue and the green with a shift in the maximum excitation of the blue component (Figure 5d). This can be due to the dissolution of green-emitting aggregates of citrazinic acid in strong basic conditions, which causes a blue shift of the C-dots emission.

**Figure 6:**
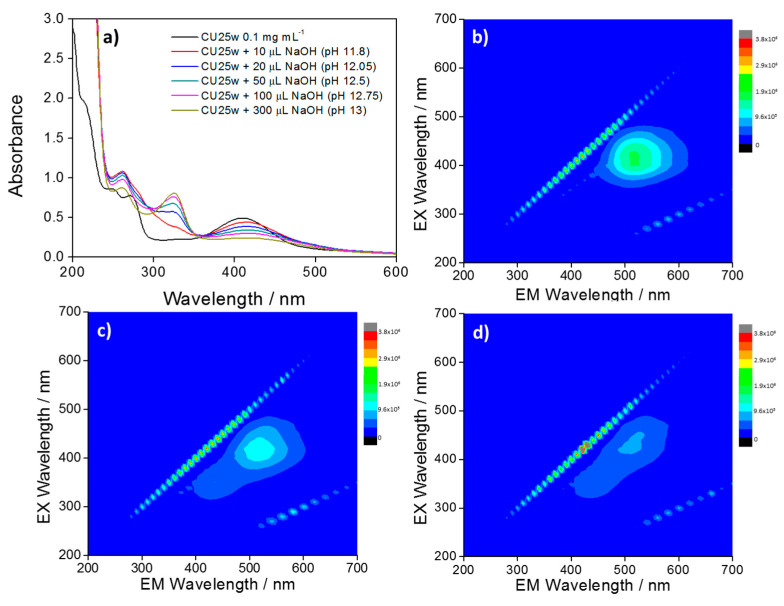
(**a**) UV–Vis spectra of CU25 C-dots (black line) and after consecutive additions of NaOH. 3D photoluminescence spectra (excitation (y-axis), emission (x-axis), intensity (false colors scale) of (**b**) CU25 C-dots in water and after addition of (**c**) 50 µL and (**d**) 100 µL NaOH. C-dots concentration = 100 mg L^−1^.

Figure 6a shows the UV–Vis spectra of CU25 dots at the increase of pH values. At high pH, the deprotonation of the fluorophores [25,29] and the subsequent formation of carbonyl groups determine the rise of the absorption band at around 328 nm, assigned to the n–π* electron transitions of C=O bonds [30]. This process affects the emission yield with a gradual quenching of the green component and the appearance of the blue one (Figure 6b–d).

DLS measurements provide new insight into the mechanism of pH-induced aggregation. Table 1 shows the C-dots hydrodynamic radii at different pH. The radius in water and NaOH solutions for CU2 and CU25 C-dots is very similar while there is a sensitive aggregation in acidic solutions, especially for the CU25 sample. The particles tend to agglomerate and to form clusters with a Gaussian distribution peaking around 0.6 μm (Figure S5).

The C-dots aggregation measured by DLS can be correlated with the AQY values summarized in Table 2. The acidic conditions cause a consistent decrease of the quantum yield, in particular for the CU25 sample. This strong quenching in emission is therefore due either to the aggregation of the fluorophores and the C-dots at low pH.

**Figure 7:**
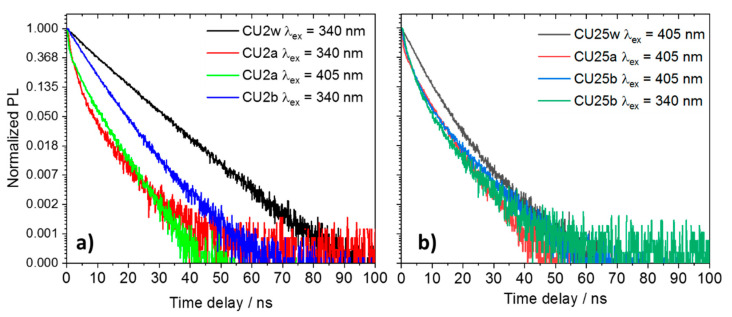
Luminescence decay profile of 100 mg L^−1^ C-dots in different aqueous solutions excited with a 340 and 405 nm laser (w = water, a = acid, b = base). (**a**) CU2 and (**b**) CU25.

Figure 7 shows the fluorescence decays of the CU2 (Figure 7a) and CU25 (Figure 7b) excited at two different wavelengths (340 and 405 nm). In general, the decay profiles can be fitted by a two-exponential law. As already reported, the different chemical environments affect the mechanism of de-excitation. The various fluorophores in the C-dots undergo protonation, followed by aggregation in acidic conditions, and deprotonation in alkaline media. In general, it is observed that the samples in water (pH ≈ 6) exhibit a longer average lifetime with respect to highly acidic and basic conditions (Table S1). In particular, due to the formation of large aggregates in acid, the non-radiative recombination channels favor a drastic reduction in lifetime. By contrast, in aqueous and alkaline solutions the C-dots show similar lifetime decays as a result of a more effective dispersion.

As shown in Figure S1, the C-dots obtained by carbonization in anhydrous conditions lack detectable crystalline structures. The characteristic lattice spacing of about 0.24 nm, corresponding to the in-plane spacing of graphite (100) [31] is not measurable in this case. Nonetheless, the optical properties of C-dots herein presented have been measured for QCDs as well [31], although they differ in structural and morphological features. This agrees with the fluorophore-like origin of the emission. In particular, the C-dots reproduce the fluorescence of molecular fluorophores and the response under extreme pH conditions. For citric acid and urea-derived C-dots, the graphitic core and surface states model does not properly explain the effect of chemical surrounding variations on the luminescence properties. Indeed, the C-dots can be considered as permeable nanostructures of carbon, which include fluorophores sensitive to the external environment. The molecular units can affect the overall luminescence signal by promoting the aggregation and disaggregation of carbonaceous superstructure through the protonation and deprotonation process.

## 4. Conclusions

C-dots prepared from citric acid and urea show a different response as a function of the pH and relative concentration of their precursors. The CU25 sample in water shows only a green component while the CU2 exhibits both blue and green emissions. The photoluminescence of CU2 and CU25 has been attributed to molecular-like fluorophores: citrazinic acid and HPPT.

In extreme acidic conditions, CU2 dots show a quenching of the blue emission while the green component remains detectable. This response, which is reversible with the pH, is explained with the formation of citrazinic acid aggregates in the C-dots structure. The aggregates emit in the green, as confirmed by the experiments on pure citrazinic acid in solution. The emission of CU25 dots, which is mainly attributed to HPPT, shows small changes in an acidic solution, indicating that the green fluorophore is less sensitive to the pH of the medium.

In extreme basic conditions, the molecular-like fluorophores are in a de-protonated form. This state favors the disaggregation of the fluorescent molecules. The CU2 C-dots show two different emission bands attributed to citrazinic acid and HPPT monomers. In contrast, the CU25 C-dots, beside the HPPT green emission, display a blue component correlated to the dissolution of citrazinic acid aggregates.

Considering the whole photoluminescence fingerprint of both CU2 and CU25, it can be inferred that the reported C-dots contain fluorophores that aggregate as a function of the dispersing medium pH. The fluorophores strongly interact with the external environment and evaluation of the emission properties in different media has to be, therefore, carefully considered. The high sensitivity to the chemical environment opens new scenarios for future applications in the field of sensing and diagnostics.

## Figures and Tables

**Table 1:** The hydrodynamic radius of CU2 and CU25 C-dots in the three aqueous solutions at different pH.

Sample	Diameter/nm
CU2/water	7.0
CU2/H_2_SO_4_	11.5
CU2/NaOH	9.5
CU25/water	6.5
CU25/H_2_SO_4_	606.9
CU25/NaOH	6.5

**Table 2:** Absolute photoluminescence quantum yield (AQY) results of C-dots dispersed in different media.

Sample	AQY
CU2/water	20.87
CU2/H_2_SO_4_	7.89
CU2/NaOH	16.08
CU25/water	13.68
CU25/H_2_SO_4_	0.59
CU25/NaOH	5.55

## Supplementary Materials

The following are available online at https://www.mdpi.com/1996-1944/13/16/3654/s1, Figure S1: Representative TEM images of (a) CU2 and (b) CU25.; Figure S2: 3D photoluminescence spectra (excitation (y-axis), emission (x-axis), intensity (false colors scale)) of citrazinic acid (a) in water and (b) in H_2_SO_4_ 10% at concentration of 10 mg L^−1^; Figure S3: PL emissions of CU2 C-dots in sulfuric acid (10%) with excitation at 350 nm (black) and 420 nm (red) at the C-dots concentrations of (a) 1 mg L^−1^ and (c) 0.1 mg L^−1^ and after neutralization with NaOH pellets with excitation at 350 nm (black) and 420 nm (red) at the C-dots concentrations of (b) 1 mg L^−1^ and (d) 0.1 mg L^−1^. * The asterisks indicate Raman vibrational modes of water; Figure S4: Light scattering analysis of CU2 and CU25 C-dots in the aqueous solutions (10 mg L^−1^) at different pH values (water = 7, H_2_SO_4_ = 1, NaOH = 14). CU2 in (**a)** water; (**b**) H_2_SO_4_ and (**c**) NaOH; CU25 in (**d**) water, (**e**) H_2_SO_4_ and (**f**) NaOH; Table S1: Decay lifetimes under excitations at 340 and 405 nm.
