# Supplementary material for: Reversible Aggregation of Molecular-Like Fluorophores Driven by Extreme pH in Carbon Dots
Source: Materials (Basel). 2020 Aug 18;13(16):3654. doi: 10.3390/ma13163654 (PMC7476021; doi:10.3390/ma13163654)
Supplement: Supplementary file 1 [file materials-13-03654-s001.pdf]

materials

MDPI

Supporting information

Reversible Aggregation of Molecular-Like Fluorophores Driven by Extreme PH in Carbon Dots

Stefania Mura, Luigi Stagi, Robert Ludmerczki, Luca Malfatti and Plinio Innocenzi *

Laboratorio di Scienza dei Materiali e Nanotecnologie, CR-INSTM, Dipartimento di Chimica e Farmacia, Università di Sassari, Via Vienna 2, 07100 Sassari, Italy; stmura@uniss.it (S.M.); lstagi@uniss.it (L.S.); ludmerczki@gmail.com (R.L.); luca.malfatti@uniss.it (L.M.)

* Correspondence: plinio@uniss.it

Received: 1 July 2020; Accepted: 13 August 2020; Published: date

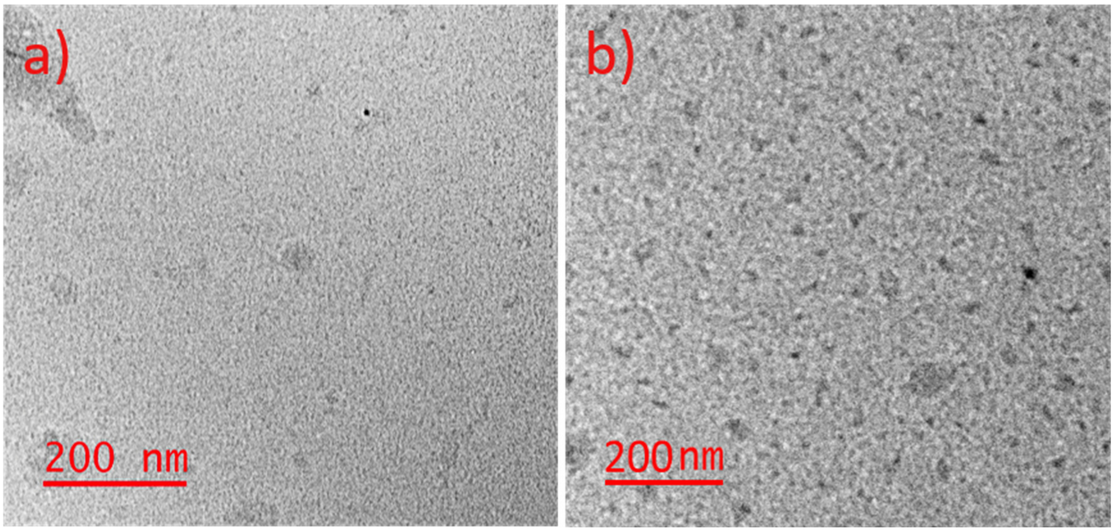

Figure S1. Representative TEM images of (**a**) CU2 and (**b**) CU25.

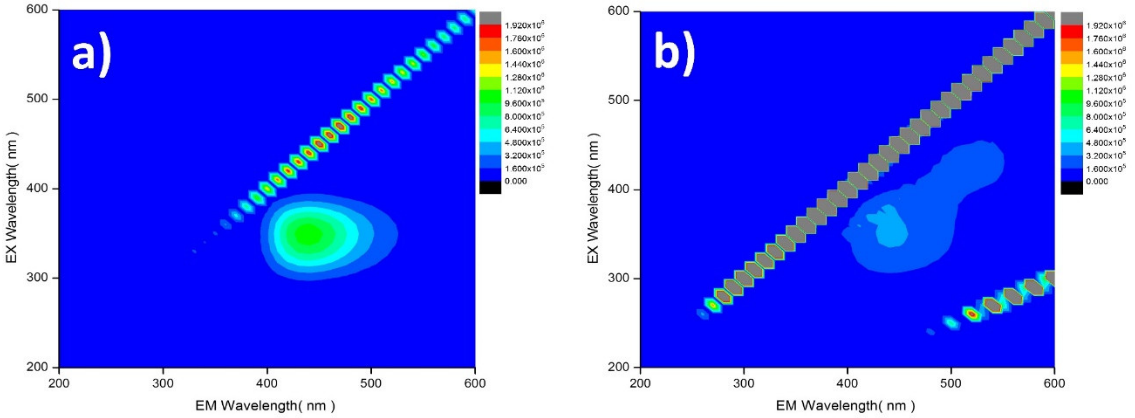

Figure S2. 3D photoluminescence spectra (excitation (y-axis), emission (x-axis), intensity (false colors scale)) of citrazinic acid (**a**) in water and (**b**) in H_2SO_4 10% at concentration of 10 mg L^{-1}.

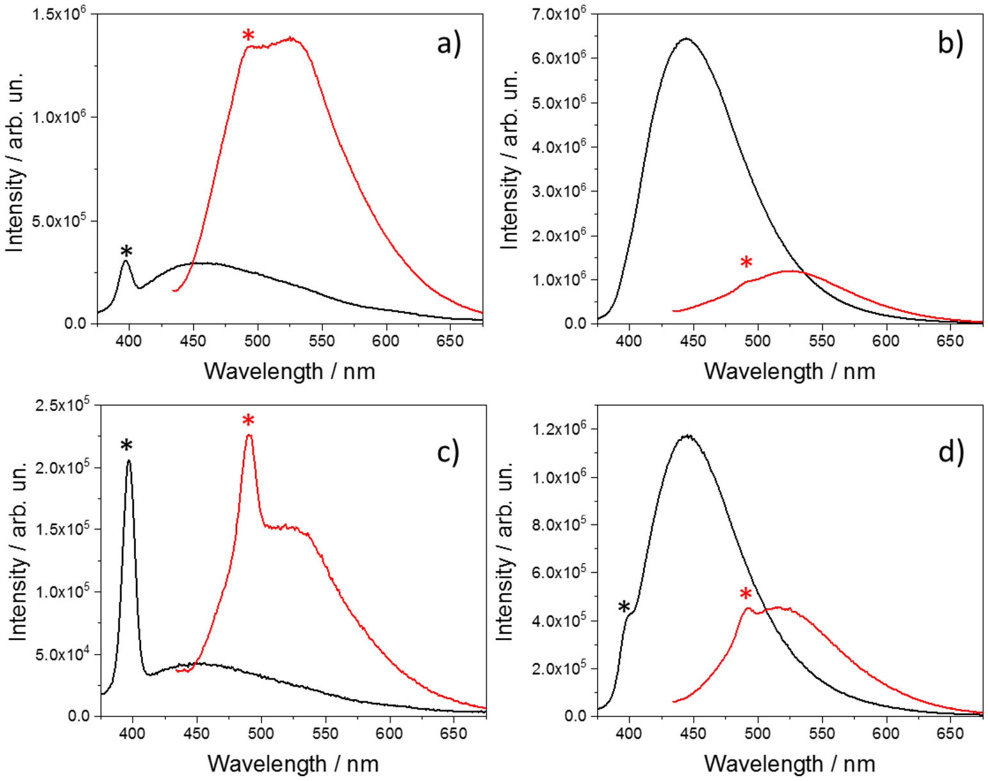

Figure S3. PL emissions of CU2 C-dots in sulfuric acid (10%) with excitation at 350 nm (black) and 420 nm (red) at the C-dots concentrations of (**a**) 1 mg L^{-1} and (**c**) 0.1 mg L^{-1} and after neutralization with NaOH pellets with excitation at 350 nm (black) and 420 nm (red) at the C-dots concentrations of (**b**) 1 mg L^{-1} and (**d**) 0.1 mg L^{-1}.The asterisks indicate Raman vibrational modes of water; Figure S4: Light scattering analysis of CU2 and CU25 C-dots in the aqueous solutions (10 mg L^{-1}) at different pH values (water = 7, H_2SO_4 = 1, NaOH = 14); * The asterisks indicate Raman vibrational modes of water.

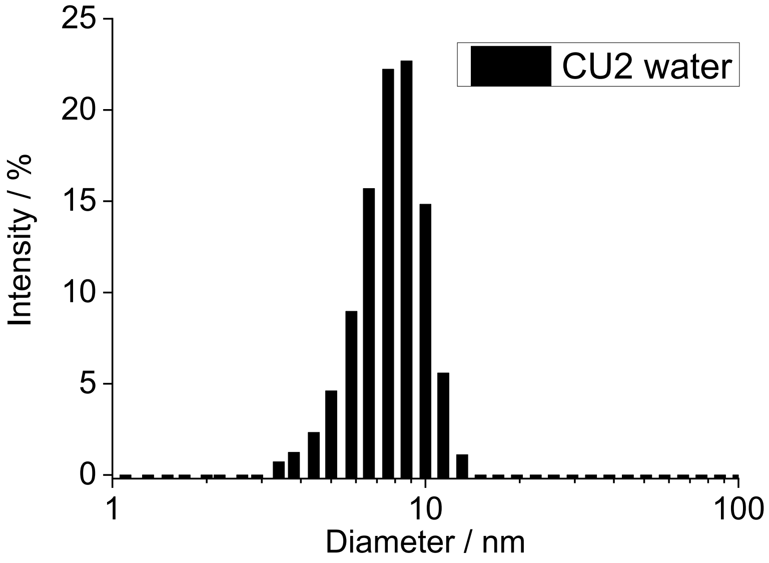

(**a**)

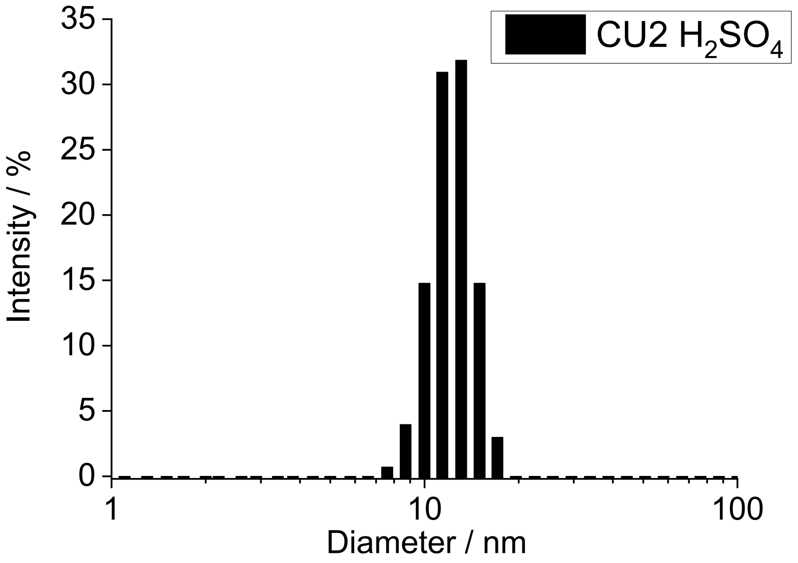
CU2 H2SO4
Intensity / %
Diameter / nm

(**b**)

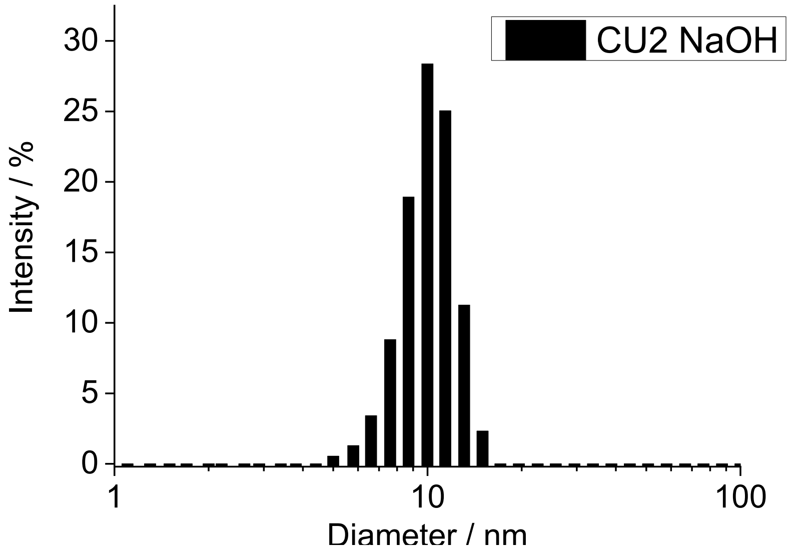
CU2 NaOH
Intensity / %
Diameter / nm

(**c**)

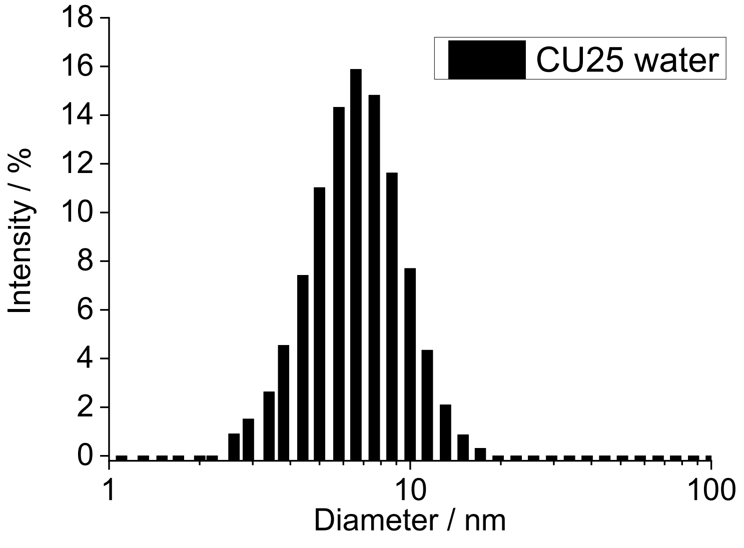
CU25 water
Intensity / %
Diameter / nm

(**d**)

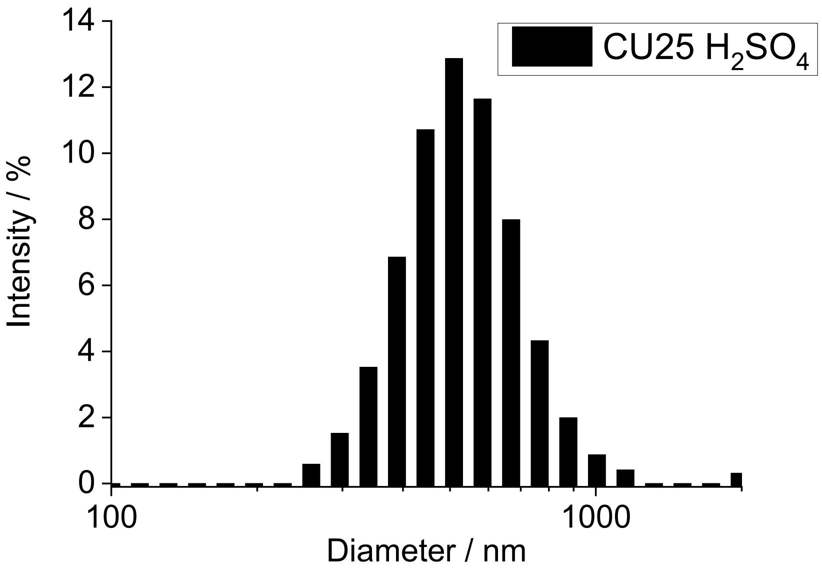

(**e**)

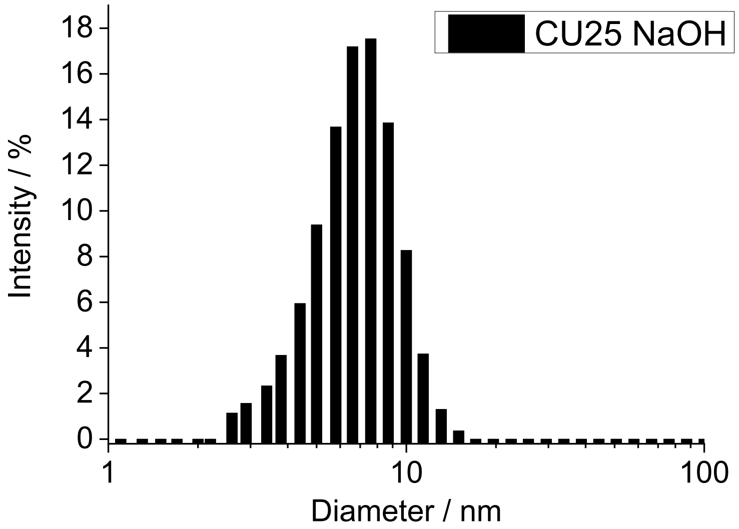

(**f**)

Figure S4. Light scattering analysis of CU2 and CU25 C-dots in the aqueous solutions (10 mg L^{-1}) at different pH values (water = 7, H_2SO_4 = 1, NaOH = 14). CU2 in (**a**) water; (**b**) H_2SO_4 and (**c**) NaOH; CU25 in (**d**) water, (**e**) H_2SO_4 and (**f**) NaOH.

Table 1. Decay lifetimes under excitations at 340 and 405 nm.

**SAMPLE**	**τ_1**	**τ_2**
CU2/water (λ_{ex} = 340 nm; λ_{em} = 420 nm)	4.7 ns	10.0 ns
CU2/H_2SO_4 (λ_{ex} = 340 nm; λ_{em} = 420 nm)	2.1 ns	7.7 ns
CU2/H_2SO_4 (λ_{ex} = 405 nm; λ_{em} = 510 nm)	2.5 ns	6.2 ns
CU2/NaOH (λ_{ex} = 340 nm; λ_{em} = 420 nm)	5.3 ns	9.1 ns
CU25/water (λ_{ex} = 405 nm; λ_{em} = 510 nm)	4.4 ns	9.1 ns
CU25/H_2SO_4 (λ_{ex} = 405 nm; λ_{em} = 510 nm)	3.2 ns	6.9 ns
CU25/NaOH (λ_{ex} = 340 nm; λ_{em} = 420 nm)	2.3 ns	7.8 ns
CU25/NaOH (λ_{ex} = 405 nm; λ_{em} = 510 nm)	2.8 ns	9.1 ns
